# Atypical presentation of cholecystitis with torsion of the gallbladder diagnosed preoperatively in an unusual location

**DOI:** 10.1259/bjrcr.20210141

**Published:** 2021-11-17

**Authors:** Sowmiya Kalyanasundaram, Suresh Fernando

**Affiliations:** 1 Radiology, Queen Elizabeth Hospital NHS Foundation Trust, Kings Lynn, Norfolk, United Kingdom

## Abstract

**Objective:**

An 87-year-old attended the emergency department with sharp upper abdominal pain, radiating to back with a pain score of 10/10. On examination, severe epigastric tenderness was noted.

Past surgical history: bilateral salphingo-oopherectomy. Repair for paraumblical hernia and right total hip replacement. No history of cholecystectomy. Inflammatory markers were raised.

Cholecystitis/gall bladder perforation was suspected and contrast CT was performed.

**Methods:**

CT abdomen and pelvis – in comparison to the previous CT scan which was done in 2018, where gall bladder was in the correct anatomical location, the gall bladder was not seen in the gall bladder fossa in the current study.

However, a gall bladder like suspicious structure was noticed within the upper abdomen to the left of midline anterior to the gastric pylorus with significant inflammatory changes.

Therefore, considering the clinical picture and CT findings, it was suggestive of acute cholecystitis with torsion of gall bladder.

**Results:**

Patient was started on i.v. antibiotics and laparoscopic assessment was carried out on the following day.

Intraoperatively, the surgeons were unable to locate the gall bladder in its normal anatomical position, but incidentally found a mass in the left upper abdomen which appeared gangrenous. This was removed and sent for histopathology.

Histology report confirmed that the specimen was gall bladder with features suggestive of pre-existing chronic cholecystitis, with recent venous infarction.

**Conclusion:**

Torsion of gall bladder is a very rare entity and if left untreated could lead to fatal sequelae of gangrene and perforation resulting in biliary peritonitis. There is evidence which suggest that torsion of gall bladder is more common in elderly females due to loss of visceral fat but the pre-operative diagnosis using imaging modalities has always been challenging. But in this particular case, the radiologist was able to make the precise diagnosis pre-operatively using the cross-sectional study of an advanced imaging modality like the CT scan with contrast which also helped the surgeons in making the decision for immediate surgery rather than planning for routine conservative management for acute cholecystitis.

The importance of cross-sectional study with intravenous contrast in diagnosing unusual presentation of gall bladder related and potentially life-threatening abdominal pathology has been highlighted in this case study. It is also evident that how imaging modalities play a significant role in altering acute management plan.

## Background

Acute gallbladder torsion remains a relatively uncommon process, usually diagnosed intraoperatively with or without the presumptive diagnosis of acute cholecystitis.^1^

Gallbladder torsion is a condition where the gallbladder twists on its long axis causing vascular compromise and as a result secondary gallbladder ischaemia or necrosis occurs. This condition has to be treated surgically, and early intervention is important, as any delay may lead to a fatal outcome.^2^ According to the literature, the mortality rate of torsion of the gallbladder is 6%, and none of the deaths occurred in patients who were diagnosed pre-operatively. It is likely that early diagnosis and intervention can reduce the morbidity and mortality associated with this condition.^3^

Unfortunately, gallbladder torsion is often overlooked since it is a rare condition, and the symptoms may initially mimic other more common gallbladder-related and upper-abdominal conditions.^4^

According to literature reviews, out of 500 only 10% of patients have been diagnosed pre-operatively.^5^

## Summary

Gallbladder torsion is a rare entity, and pre-operative diagnosis is especially challenging irrespective of advancements in modern technology. Untreated torsion of the gallbladder can lead to fatal sequelae of gangrene and perforation, resulting in biliary peritonitis.^4,6^ This case report highlights how the study of precise cross-sectional and contrast CT can help with early diagnosis and appropriate management.

An 87-year-old female patient was admitted with acute upper-abdominal pain with raised inflammatory markers. On a contrast-enhanced CT scan of the abdomen and pelvis, the gallbladder was not visible in the gallbladder fossa. However, a suspicious gallbladder-like structure with significant inflammatory changes was noted within the upper abdomen to the left of the midline. The diagnosis made by the radiologist was acute cholecystitis with torsion of the gallbladder. Based on CT findings, a laparoscopic intervention was planned, and the abnormal-looking ischemic mass was surgically removed. Histology of the specimen confirmed the CT findings.

## Case presentation

An 87-year-old lady attended the emergency department with sharp upper-abdominal pain radiating to the back, with a pain score of 10/10. A clinical examination revealed severe epigastric tenderness.

The patient’s past surgical history included a bilateral salphingo-oopherectomy for an ovarian cyst, a para-umbilical hernia repair and a right total hip replacement. The patient had no history of cholecystectomy. Blood reports revealed raised inflammatory markers (CRP-74), normal liver function and an amylase reading of 79.

Cholecystitis with or without complications was suspected, and further investigation with a contrast-enhanced CT scan of the abdomen was performed. Considering patient’s presenting symptom and severity, CT was more appropriate and feasible option compared to Ultrasonography to rule out acute abdomen.

## Differential diagnosis

Acute cholecystitis

Acute pancreatitis

Gastritis or gastric perforation

## Investigations/imaging: CT scan of the abdomen and pelvis

**Figure 1.**
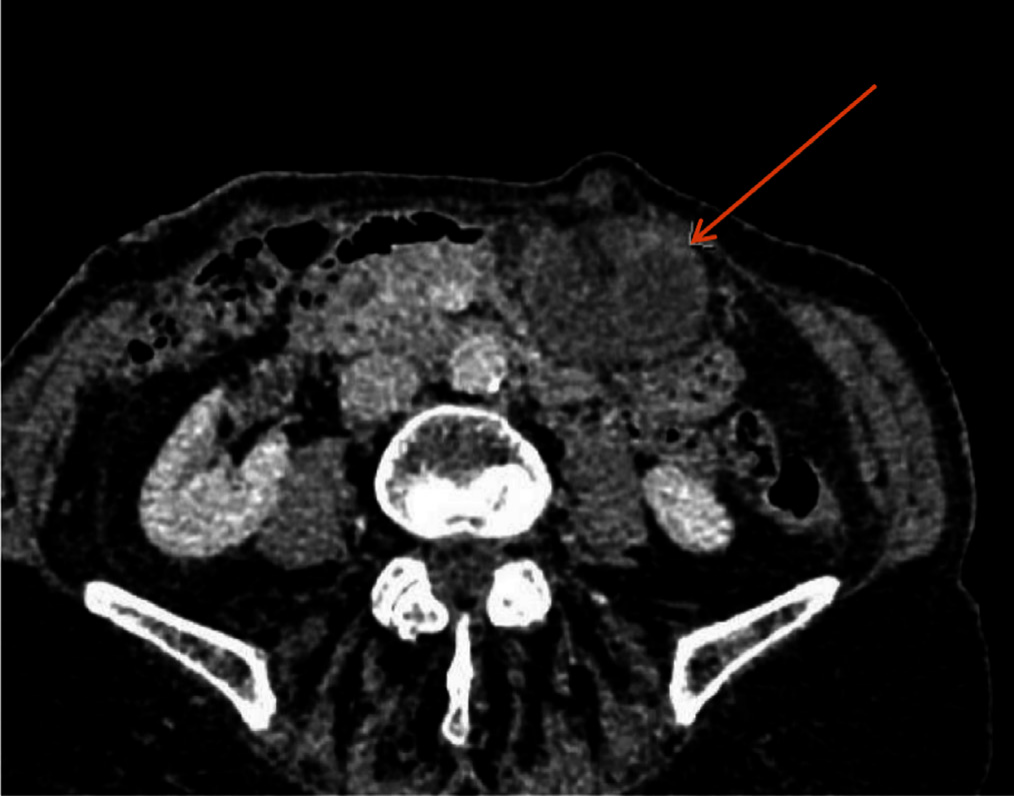
Computed Tomography: Coronal View Coronal view showing the suspected gallbladder with signs of torsion

**Figure 2.**
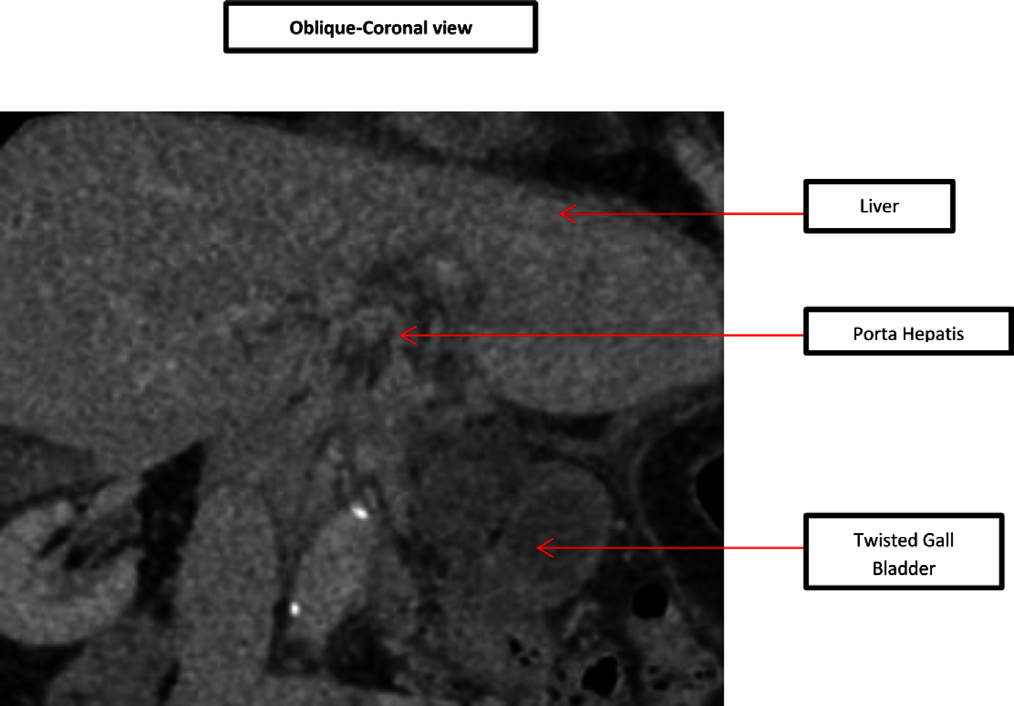
CTscan-Oblique Coronal view showing twisted gallbladder

**Figure 3.**
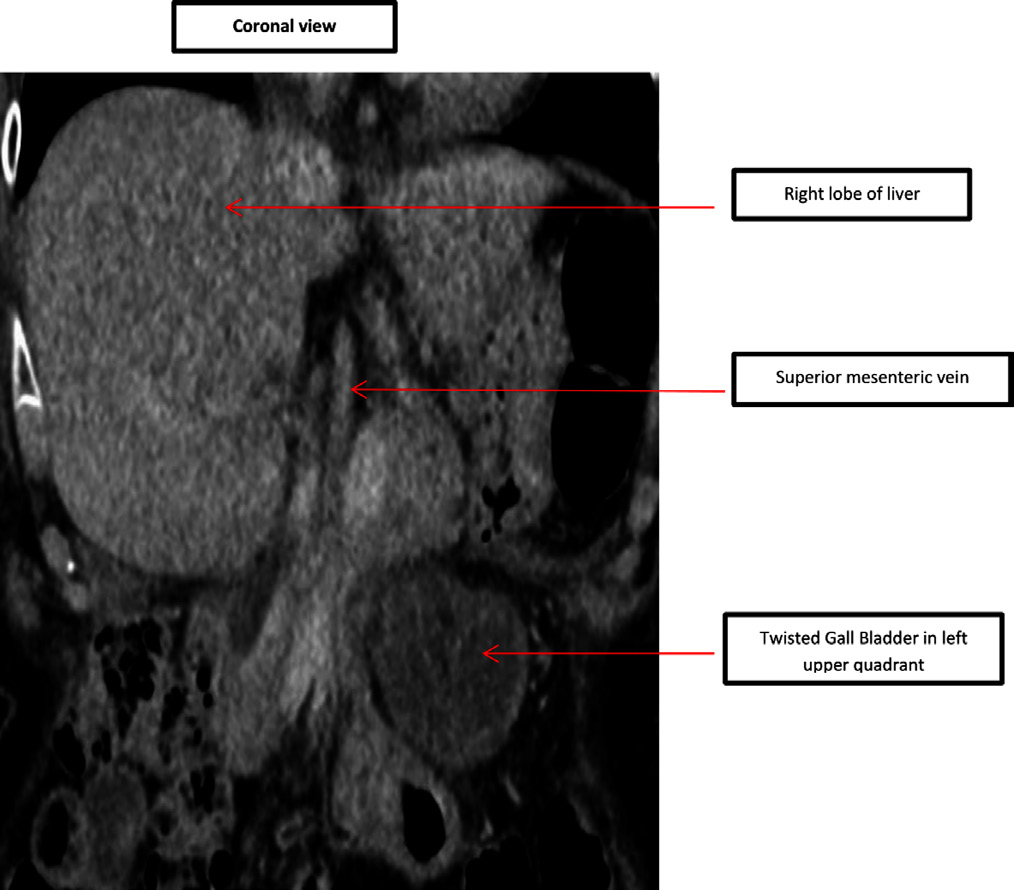
CT scan - Coronal view demonstrating twisted gall bladder in left upper quadrant

**Figure 4.**
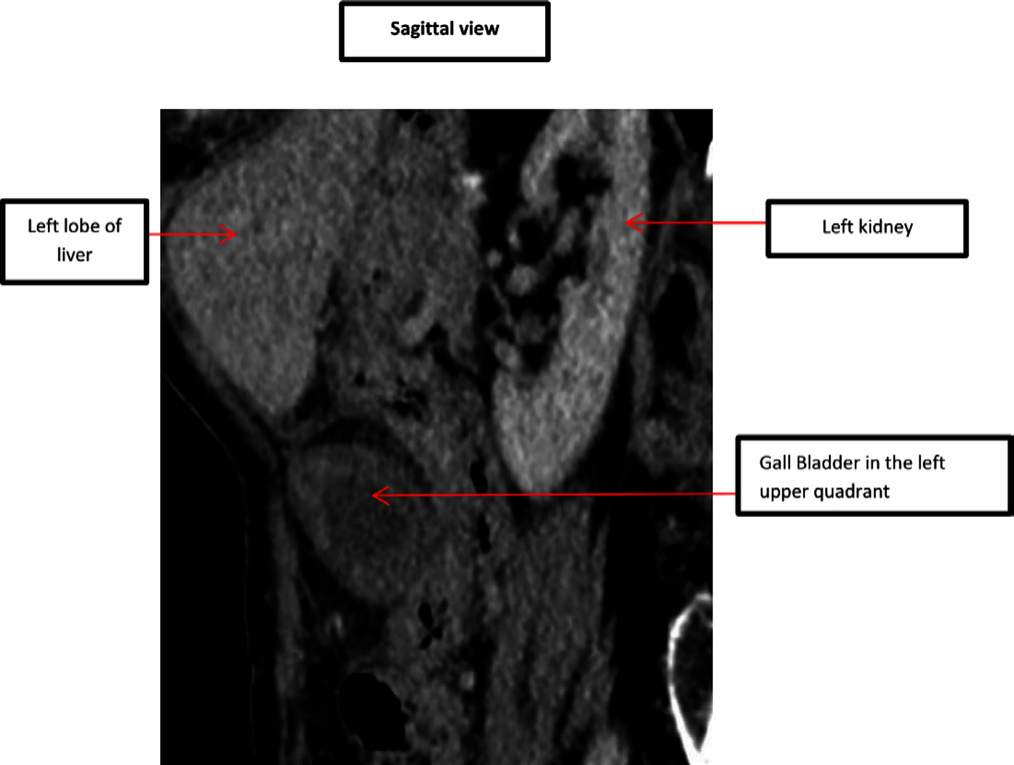
CT scan - Sagittal view showing gall bladder in the left upper quadrant

**Figure 5.**
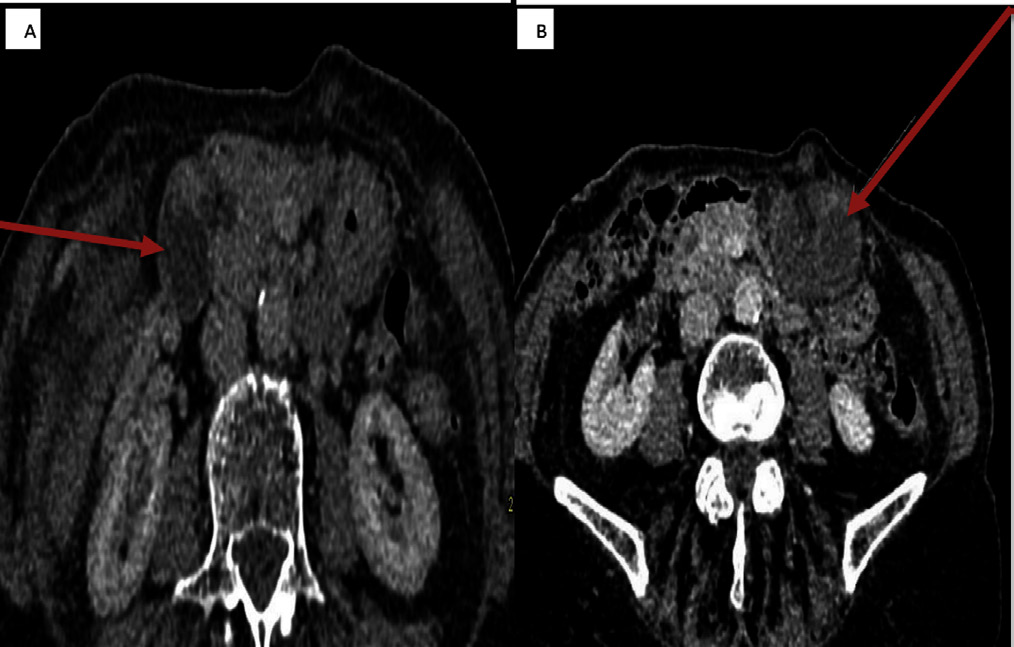
(A) Axial view CT -2018 showing Gall bladder in gall bladder fossa (B) CT -2020 Axial view demonstrating the gallbladder-like structure with evidence of ischaemia on the left side

In comparison to the previous CT scan that was carried out in 2018, where the gallbladder was in the correct anatomical location, the gallbladder was not seen in the gallbladder fossa in the current study. However, a gallbladder-like structure was noticed within the upper abdomen to the left of the midline, anterior to the gastric pylorus, with significant inflammatory changes(Figure 1) and evidence of wall ischemia Figure 2. The gall bladder measured 65 × 25 mm and was mildly distended.Figure 3 There were no signs of calculi in the gall bladder on the CT scan. As it was an unusual presentation where gall bladder was not visualized in the normal anatomical location,(Figure 4) the cystic duct could not be delineated and the swirl sign which is considered one of the strong indicators for the diagnosis of torsion of gall bladder could not be appreciated. Although, other signs such as gall bladder distension, absence of calculi, change in anatomical position of gall bladder(Figure 5) and evidence of ischemia cumulatively helped in the diagnosis of gall bladder torsion in this case.

Moderate sigmoid diverticular disease was also noted, and the appendix appeared normal. Therefore, considering the clinical picture and CT findings, the final diagnosis was acute cholecystitis with torsion of the gallbladder.

## Treatment

The patient was started on i.v. antibiotics, and an emergency laparoscopic assessment was carried out to investigate possible gallbladder torsion. Intraoperatively, the surgeons were unable to locate the gallbladder in its normal anatomical position but found a mass in the left upper abdomen, which appeared gangrenous. This was removed and sent for histopathology. The histology report confirmed that the specimen was the gallbladder with features suggestive of pre- existing chronic cholecystitis with recent venous infarction.

## Outcome

Following surgery, the patient improved clinically and repeat bloods revealed a decrease in the level of inflammatory markers (CRP-49). Therefore, the patient was discharged home after a few days.

## Discussion

Gallbladder torsion is usually seen in the elderly and frail females. Age-related changes in the supporting tissue of the gallbladder increase the risk of twisting.

The mesentery supports and supplies organs, such as the gallbladder. When there is decrease in the amount of visceral fat or in case of anatomical variants in the mesentery, increases the gall bladder mobility and that is usually called as ‘floating gall bladder’. The diagnosis of gallbladder torsion can be made with investigations, such as an abdominal ultrasound or CT scan.^8^ It is not uncommon for the diagnosis to be made intraoperatively.

Pre-operative diagnosis continues to be a major challenge, with only 10 in 500 cases reported in the literature diagnosed with pre-operative imaging; the remainder were found intraoperatively.^5^ Consequently, a delay in diagnosis can have devastating patient outcomes. Therefore, a high index of suspicion for gallbladder torsion is necessary in the outlined patient demographic, as symptoms mimic acute cholecystitis.^9^

CT protocol used in this case: single portal venous phase post-contrast image acquisition of the abdomen and pelvis (Omnipaque 350 80 ml) on Toshiba Prime Acquilion scanner with multiplanar reconstruction.

An abnormal location of the gallbladder, fluid collection in the gallbladder fossa, a shift in the axis of the gallbladder from vertical to horizontal, a hyperenhancing cystic duct to the right of the gallbladder, swirl sign along with features of acute inflammation of gall bladder wall can be seen on a CT scan in case of gall bladder volvulus. The gallbladder will be more distended in torsion than in acute cholecystitis.

**Figure 6.**
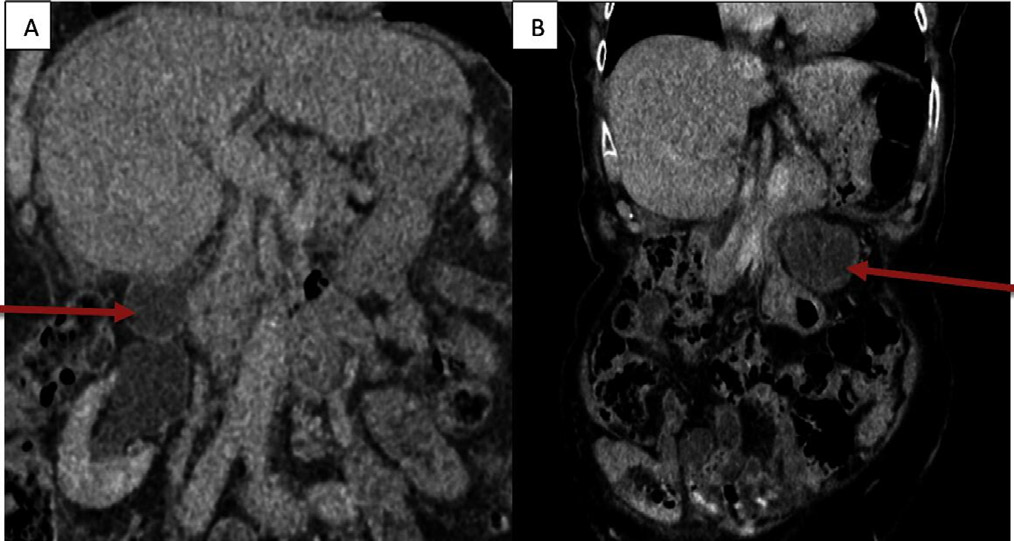
Comparison images:CT scan in 2018 and CT scan during admission (2020) A – Coronal oblique reconstruction: CT 2018 showing normal location of gall bladder Image B: CT 2020: Coronal view showing the suspected gallbladder with signs of torsion

The swirl sign (the vascular pedicle and the surrounding fat dive in and form a mini “swirl” appearance) which is considered one of the strong indicators for diagnosing torsion of gall bladder could not appreciated in this case but other signs such as gall bladder distension, absence of calculi, change in anatomical position of gall bladder and evidence of ischemia Figure 6cumulatively helped in the diagnosis of gall bladder torsion.

The treatment for gallbladder torsion is an emergency cholecystectomy.^2,10^

If the condition is diagnosed early and if surgical intervention is carried out immediately, then the outcome is good. However, severe cases can be life-threatening without early intervention.

## Learning points

Evidence suggests that gallbladder torsion is more common in elderly females due to the loss of visceral fat and elasticity. However, pre-operative diagnosis using imaging modalities has always been challenging.

The diagnosis of gall bladder torsion can be made with the following CT findings: distended gall bladder, change in axis or position, ischemic gall bladder, “beak” and “swirl” appearance at the gall bladder neck.

In this case, the radiologist was able to make a precise diagnosis of gallbladder torsion pre-operatively using a contrast-enhanced CT scan. This influenced the surgeons’ decision to opt for emergency surgery rather than conservative management with interval cholecystectomy for acute cholecystitis.

The importance of a cross-sectional study with intravenous contrast in diagnosing the unusual presentation of gallbladder-related and potentially life-threatening abdominal pathology has been highlighted in this case study. It is also evident that imaging modalities play a significant role in altering the acute management plan.

Advancement in the last few years in the field of radiology especially in imaging techniques has enabled the pre-operative diagnosis of one-quarter of patients with gallbladder torsion. With early diagnosis and prompt surgical intervention, gall bladder torsion has excellent prognosis.
